# Editorial: Incretin agonists in the treatment of obesity

**DOI:** 10.3389/fendo.2023.1265826

**Published:** 2023-08-09

**Authors:** Xiaodong Sun, Miaofen G. Hu, Lixin Li

**Affiliations:** ^1^ Department of Endocrinology and Metabolism, Affiliated Hospital of Weifang Medical University, Weifang, China; ^2^ Clinical Research Center, Affiliated Hospital of Weifang Medical University, Weifang, China; ^3^ Department of Medicine, Division of Hematology and Oncology, Tufts Medical Center, Boston, MA, United States; ^4^ Central Michigan University, College of Health Professions, Mount Pleasant, MI, United States

**Keywords:** obesity, GLP-1, GLP-1R agonists, liraglutide, semaglutide, GIP, Dual GIP/GLP-1R agonists, incretins

Obesity has emerged as a critical global public health concern over the past decade. Studies have demonstrated that even a modest weight reduction significantly decreases the risk of developing obesity-related complications and chronic disease (1). However, current anti-obesity medications have certain limitations due to concerns about their safety and efficacy (2, 3). Recently, emerging evidence indicates incretin-based therapies, such as glucagon-like peptide-1 receptor (GLP-1R) agonists and novel co-agonists that activate both the GLP-1R and the glucose-dependent insulinotropic polypeptide receptor (GIPR) have shown promising effects in treating obesity (4). These incretin agonists reduce body weight through various pathways, including suppressing food intake, delaying gastric emptying, and activating brown fat (5, 6). However, the exact underlying mechanism of incretin-based therapies in treating obesity, as well as their long-term safety and efficacy, remains uncertain. Further evidence is needed to establish their long-term safety and efficacy. This Research Topic features a collection of 2 original research articles and 3 review articles that compile and disseminate recent evidence and knowledge regarding the therapeutic effects and the underlying mechanisms of these incretin agonists in the treatment of obesity.

Exenatide, the first clinically used GLP-1 receptor agonist, has shown potential therapeutic benefits in metabolic disorders. In the original research article, Bai et al. highlights the potential of exenatide as a therapeutic intervention for improving obesity related hepatic insulin resistance. They demonstrate that exenatide inhibit the secretion of adipokines from adipose tissues, specifically, when human subcutaneous adipose tissue (SAT) and visceral adipose tissue (VAT) were co-cultured with HepG2 cells, the inhibitory effect was particularly evident in VAT. Furthermore, they show that exenatide improves hepatic insulin sensitivity through regulating hepatic IRS2/PI3K/Akt2 insulin signaling pathway. This is achieved by up-regulating IRS2, PI3K-p85, and p-Akt2, while down-regulating p-IRS2 (S731). This study highlights the differences in adipokine secretion between visceral VAT and SAT and emphasizes the role of exenatide in regulating adipokine profiles. These findings underscore the importance of considering regional fat distribution and adipokine profiles when targeting therapeutic strategies for obesity-related metabolic disorders.

Semaglutide, a novel GLP-1 analogue, has shown promising potential in promoting weight loss. Moreover, emerging evidence suggests that semaglutide may provide cardiometabolic benefits in individuals with obesity. In a meta-analysis comprising 13 randomized controlled trials, Zhang et al. examined the varying therapeutic effects of semaglutide on weight control under different administration circumstances. The study indicates that higher weekly dosages of 2.0 mg or more, longer treatment durations, and severe baseline BMI contribute to more pronounced weight loss effects. Furthermore, the importance of incorporating lifestyle interventions alongside semaglutide treatment is highlighted. The study also reveals additional benefits of semaglutide treatment on cardiometabolic profiles. Overall, this study indicates the subcutaneous semaglutide emerges as a promising and safe therapeutic option for weight control in overweight or obese individuals, with the potential to improve health outcomes.

Zhu and Chen conducted an animal study that revealed the favorable impact of semaglutide on adipose tissue and lipid metabolism in obese mice. The administration of semaglutide for 12 weeks led to significant reductions in the accumulation of VAT, improved plasma lipid levels, and improved glucose intolerance and enhanced insulin sensitivity. A proteomic analysis of epididymal white adipose tissue (eWAT) revealed expression of proteins involved in lipid metabolism. These findings suggest that semaglutide exerts regulatory effects on lipid uptake, storage, and lipolysis, particularly in eWAT. These findings make semaglutide a promising therapeutic option for combating metabolic disorders associated with obesity.

In a comprehensive review, Wang et al. highlights both short-acting and long-acting GLP-1R agonist and their potential as a therapeutic approach for obesity prevention and management. These GLP-1R agonists have shown promising results in both animal experiments and clinical trials. The comprehensive understanding of the effects on obesity treatment presented in this review can serve as a guidance framework for future biomedical research and clinical practice. This knowledge can contribute to the development of improved treatments for individuals with obesity.

Recently, incretin-based therapies, which involve the coactivation of both GIPR and GLP-1R, have garnered interest due to their combined benefits on weight loss and glycemial control. A review by Zaffina et al. provided a comprehensive summary of tirzepatide, the first medication in this class. The review highlighted the potential therapeutic benefits of tirzepatide, including improved glycemic control, increased insulin sensitivity, enhanced lipid metabolism, and reduced body weight. This review also emphasized the physiological mechanisms of the dual GIPR/GLP-1R agonist in weight management. By simultaneously targeting both GIP and GLP-1 receptors, tirzepatide offers a more comprehensive approach in treating diabetes and obesity while potentially minimizing adverse effects compared to selective GLP-1R agonists, underscoring the promise of tirzepatide as a novel anti-obesity treatment and shedding light on its synergistic mechanisms and potential in improving patient outcomes.

Overall, the escalating prevalence of obesity presents a pressing global health challenge. The articles presented with the Research Topic highlight the crucial role of various incretin agonists in weight reduction and improvement of cardiometabolic profiles. Nevertheless, ongoing research is vital to refine and expand the arsenal of anti-obesity therapies. Future investigations should focus on three main areas: long-term safety, efficacy, and the identification of patient-specific factors that may have an impact on treatment response.

## Author contributions

XS: Conceptualization, Funding acquisition, Writing – original draft, Writing – review & editing. MH: Conceptualization, Data curation, Supervision, Validation, Writing – review & editing. LL: Conceptualization, Data curation, Supervision, Validation, Writing – review & editing.
